# Cross-Cultural Differences and Clinical Presentations in Burning Mouth Syndrome: A Cross-Sectional Comparative Study of Italian and Romanian Outpatient Settings

**DOI:** 10.3390/jcm14165805

**Published:** 2025-08-16

**Authors:** Claudiu Gabriel Ionescu, Gennaro Musella, Federica Canfora, Cristina D’Antonio, Lucia Memé, Stefania Leuci, Luca D’Aniello, Ioanina Parlatescu, Lorenzo Lo Muzio, Michele Davide Mignogna, Serban Tovaru, Daniela Adamo

**Affiliations:** 1 Department of Ethics and Academic Integrity, Carol Davila University of Medicine and Pharmacy, 050474 Bucharest, Romania; claudiu.ionescu@umfcd.ro; 2 Department of Clinical and Experimental Medicine, University of Foggia, 71122 Foggia, Italy; lorenzo.lomuzio@unifg.it; 3 Department of Neuroscience, Reproductive and Odontostomatological Sciences, University of Naples Federico II, 80138 Naples, Italy; federica.canfora@unina.it (F.C.); stefania.leuci@unina.it (S.L.); mignogna@unina.it (M.D.M.); d.adamo@unilink.it (D.A.); 4 Department of Health Sciences, School of Dentistry, Magna Graecia University of Catanzaro, 88100 Catanzaro, Italy; cristina.dantonio@studenti.unicz.it; 5 Department of Life Sciences, Health and Health Professions, Link Campus University, Via del Casale Di San Pio V 44, 00165 Rome, Italy; l.meme@unilink.it; 6 Department of Economics and Statistics, University of Naples Federico II, 80126 Naples, Italy; luca.daniello@unina.it; 7 Faculty of Dentistry, Carol Davila University of Medicine and Pharmacy, 050474 Bucharest, Romania; ioanina.parlatescu@umfcd.ro (I.P.); serban.tovaru@gmail.com (S.T.)

**Keywords:** Burning Mouth Syndrome, chronic pain, cross-cultural comparison, facial pain, perceptual disorders, sleep disorders

## Abstract

**Background/Objectives**: Burning Mouth Syndrome (BMS) is a chronic orofacial pain disorder characterized by persistent intraoral burning sensations without visible mucosal lesions. Although its biopsychosocial complexity is increasingly recognized, cross-cultural comparison data remain limited. **Methods**: This cross-sectional study assessed 60 patients with BMS (30 Italian, 30 Romanian) who underwent standardized clinical, psychological, and sleep evaluations. Data collected included sociodemographics, clinical characteristics, diagnostic history, comorbidities, and symptomatology. The assessment tools used included the Numeric Rating Scale (NRS), Short Form of the McGill Pain Questionnaire (SF-MPQ), Hamilton Anxiety Rating Scale (HAM-A), Hamilton Depression Rating Scale (HAM-D), Pittsburgh Sleep Quality Index (PSQI), and Epworth Sleepiness Scale (ESS). Statistical comparisons were conducted using Mann–Whitney U and Fisher’s exact tests with Bonferroni correction. **Results**: No significant differences were observed in age, sex, or body mass index. Italian patients had fewer years of education (*p* = 0.001), higher pain intensity (NRS, *p* < 0.001), poorer sleep quality (PSQI, ESS, *p* = 0.001), and more frequent pre-existing sleep disorders (*p* < 0.001). Romanian patients showed higher levels of anxiety (HAM-A, *p* < 0.001), longer diagnostic delays (*p* = 0.002), and more dysesthetic or perceptual symptoms, including tingling and oral dysmorphism (*p* < 0.05). Stressful events before onset were more common among Romanians (*p* < 0.001), while Italians more often received a correct diagnosis at first consultation (*p* = 0.005). **Conclusions**: This first cross-national comparison of BMS in Western and Eastern Europe shows that cultural, healthcare, and clinician education differences can shape symptom profiles, comorbidities, and diagnostic delays, underscoring the need for personalized, country-specific management strategies.

## 1. Introduction

Burning Mouth Syndrome (BMS) is a chronic pain condition characterized by a burning or dysesthetic sensation, recurring daily for more than two hours per day over more than three months, without clinically evident causative lesions. The International Classification of Orofacial Pain (ICOP, 2020) categorizes it as a neuropathic pain disorder, reflecting dysfunction of the peripheral or central nervous system [1]. The condition is also referred to by other names such as glossodynia, burning tongue, stomatodynia, or oral dysesthesia [2]. Epidemiologic studies estimate that the prevalence of BMS is up to 8% in clinical settings and approximately 1.73% in the general population [3]. Most often occurring in postmenopausal women, usually between three years prior to and twelve years following the onset of menopause, it is 2.5 to 7 times more common in women. In a significant recent meta-analysis, a subgroup analysis revealed that the prevalence was higher among people over 50 (3.31%) than among those under 50 (1.92%) [4,5]. The pathophysiology of BMS is multifactorial, involving both peripheral and central nervous system dysfunctions [6]. Peripheral mechanisms include trigeminal small fiber neuropathy, contributing to altered thermal and pain sensitivity. Central mechanisms, such as impaired pain processing and central sensitization, also play a key role [7]. Neuroimaging studies show abnormalities in brain regions involved in pain modulation and emotional regulation [8,9]. These findings suggest that BMS lies at the interface between somatosensory dysfunction and emotional dysregulation [10]. BMS has thus been increasingly framed as a nociplastic pain disorder, characterized by altered nociception without evident tissue damage or nerve lesions [11]. According to the International Association for the Study of Pain (IASP), nociplastic pain arises from altered nociception without clear evidence of tissue damage activating nociceptors or a lesion of the somatosensory system [12]. This evolving perspective acknowledges that many BMS patients exhibit central sensitization phenomena and psychological factors, including anxiety, depression, sleep disturbance, and maladaptive coping strategies, that amplify symptoms, contribute to chronicity, and worsen quality of life [10,13]. Similar associations between pain severity, sleep quality, and life satisfaction have been described in other chronic orofacial pain conditions, reinforcing the importance of a multidimensional approach [14]. In addition to the hallmark burning sensation, patients frequently report a constellation of dysesthetic and perceptual symptoms, including xerostomia, dysgeusia, tingling, oral dysmorphism, and foreign body sensations, which reflect the complex somatosensory alterations characteristic of this disease [15].

BMS is clinically heterogeneous, with variations in symptom location, pattern (e.g., bilateral vs. unilateral), and intensity [16]. Although often of spontaneous onset, BMS may be triggered by dental procedures, medications, or stressful life events [4,17]. Symptoms commonly worsen in the evening and improve with eating; however, in long-standing disease, these circadian and meal-related fluctuations may be less pronounced or absent.

Interestingly, many patients report symptom relief while eating. This phenomenon has been attributed to increased salivary flow during mastication, which may transiently buffer mucosal sensitivity or restore a sense of oral lubrication [18].

This heterogeneity of BMS presentation can complicate early recognition, as diagnosis remains one of exclusion of other local or systemic conditions [19].

Emerging evidence suggests that sociocultural factors, such as low levels of education and socioeconomic status, as well as systemic and psychological comorbidities, can significantly shape BMS presentation and patient experiences. An Italian study reported an average diagnostic delay approaching 30 months, with patients consulting multiple specialists and frequently receiving misdiagnoses such as stomatitis or candidiasis [4], underscoring limited clinician awareness. Recent Romanian studies have highlighted distinctive psychosocial and clinical features among BMS patients, including worse sleep quality, reduced verbal fluency, higher depressive symptoms, and strong associations with somatization [20]. Conversely, an Italian investigation identified a high prevalence of obsessive-compulsive symptoms and personality traits, particularly in domains such as ordering and obsessing [21], which have been linked to perfectionism, cognitive rigidity, and maladaptive coping strategies that may affect pain modulation and treatment response. Furthermore, higher rates of hypertension and dyslipidemia have been observed in Romanian cohorts compared to Italian BMS patients [22].

Despite these findings, cross-cultural comparisons using harmonized methodologies remain scarce. To our knowledge, no study has systematically compared BMS cohorts from Western and Eastern Europe to examine how healthcare systems, patient journeys, and cultural contexts influence disease expression and diagnosis. To address this gap, we conducted a comparative study of Italian and Romanian BMS patients, assessing sociodemographic variables, diagnostic delays, symptom profiles, comorbidities, and psychological features with a standardized protocol. Exploring temporal, phenomenological, and cross-cultural variations may provide valuable insights into the biological underpinnings and healthcare-related determinants of BMS. This study represents the first cross-national, harmonized comparison of BMS cohorts from both Western and Eastern Europe, filling a critical gap in the literature. By going beyond previous single-country investigations, it sheds new light on how cultural context and healthcare infrastructure profoundly influence symptom expression, psychological burden, and diagnostic accuracy. Based on the hypothesis that Italian and Romanian patients may differ in their clinical and psychological profiles due to disparities in healthcare systems and sociocultural contexts, our aim was to generate evidence that could inform more culturally sensitive approaches to diagnosis and management, and improve the international comparability of research on BMS.

## 2. Materials and Methods

### 2.1. Study Design and Participants

This cross-sectional comparative study included patients diagnosed with BMS according to ICOP-2020 [1], who were consecutively recruited between January 2023 and February 2025 from two centers: the Oral Medicine Unit of the University of Naples “Federico II” in Italy and an outpatient clinic in “Carol Davila” University, Bucharest, Romania. The diagnosis was established by two experienced oral medicine specialists in each participating center.

This study was conducted in accordance with the Declaration of Helsinki and was approved by the Institutional Review Boards of both participating institutions: the Ethics Committee of Naples Federico II University (Approval Number: 251/19, 20 February 2019) and the Ethics Committee of “Carol Davila” University of Medicine and Pharmacy (Approval Number: 36988/2022, 29 November 2020). All patients provided written informed consent before participation. The study design, conduct, and reporting adhered to the Strengthening the Reporting of Observational Studies in Epidemiology (STROBE) guidelines [23].

The eligibility criteria were as follows:

- Patients experiencing oral burning symptoms lasting for more than 2 h per day, occurring daily, and persisting for more than 3 months, without any clinical mucosal alterations.
- Patients with normal blood test results, including complete blood count, blood glucose levels, glycated hemoglobin, serum iron, ferritin, and transferrin.
- Patients not receiving treatment with psychotropic drugs.

The exclusion criteria were as follows:

- Patients with diseases that could be identified as causative factors for BMS.
- Pregnant or childbearing patients.
- Patients unable to comprehend the questionnaires.
- Patients with a history of psychiatric, neurological, or organic brain disorders.
- Patients with a history of alcohol or substance abuse.
- Patients receiving systemic drugs that could be associated with oral symptoms.
- Patients with a history of gastroesophageal reflux disease (GERD) without stable proton pump inhibitor (PPI) therapy for at least 3 years or with recent reflux episodes.
- Patients whose symptoms could be temporally correlated with the introduction of a new medication, as medication-related side effects are expected to occur soon after treatment initiation or after changes in dosage or type.
- Patients diagnosed with Obstructive Sleep Apnea Syndrome (OSAS).

### 2.2. Clinical Assessment

A structured interview and clinical examination were performed for all participants by an oral medicine specialist (DA and ST) and a psychiatric evaluation (GP and CI).

The following demographic data were collected: age, sex, body mass index (BMI), years of education, marital status (single, married, divorced, widowed), employment status (employed, unemployed, retired), smoking status (smokers/never smokers, frequency: <5 cigarettes/day; 5–10 cigarettes/day; 10–15 cigarettes/day; >15 cigarettes/day; and the use of e-cigarettes, or heat-not-burn tobacco products), alcohol intake (drinkers/non-drinkers and frequency using a cut-off of 14 units/week), and physical activity (yes/no). Clinical characteristics of BMS were assessed, including disease duration, number of clinicians consulted before reaching a diagnosis, referral pathway, and whether the correct diagnosis was made at the first consultation. Oral symptoms were evaluated for the presence and type of dysesthetic or perceptual symptoms reported by the patients, including burning sensation, dysgeusia, tingling, allodynia, and oral dysmorphism. The worst symptom as perceived by each patient was recorded. Additionally, the anatomical distribution of symptoms within the oral cavity, the localization pattern, and the circadian pattern of symptom fluctuation were documented. The presence of systemic comorbidities was documented, and the Age-Adjusted Charlson Comorbidity Index (AACCI) [24,25] was calculated. The use of systemic medications was also recorded.

#### Psychological and Sleep Assessment

Pain intensity was assessed using a 10-point Numeric Rating Scale (NRS; 0 = no pain, 10 = worst imaginable pain) [26], displayed with numbers, with patients rating their average pain over the past week.

Pain quality was assessed using the Short-Form McGill Pain Questionnaire (SF-MPQ), which evaluates sensory, affective, and evaluative dimensions of pain. Each descriptor is rated on a scale from 0 (none) to 3 (severe), and scores are calculated by summing the ratings across the 15 descriptors, resulting in a total pain rating index ranging from 0 to 45.

Depressive symptoms were evaluated using the Hamilton Depression Rating Scale (HAM-D), where scores greater than 7 indicate clinically relevant depression. Specifically, scores of 7–17 reflect mild depression, 18–24 indicate moderate depression, and scores above 24 denote severe depression [27,28]. Anxiety was assessed with the Hamilton Anxiety Rating Scale (HAM-A), which captures both somatic and psychic dimensions of anxiety. Each item is rated on a scale from 0 to 4, yielding a total score that classifies anxiety severity: scores below 17 indicate mild anxiety, 18–24 correspond to moderate anxiety, and scores above 25 suggest moderate to severe anxiety [29,30].

Sleep quality was assessed using the Pittsburgh Sleep Quality Index (PSQI), a 19-item self-report questionnaire that evaluates multiple aspects of sleep over the previous month. A global score exceeding 5 is indicative of poor sleep quality [31]. Daytime sleepiness was measured with the Epworth Sleepiness Scale (ESS), a self-administered tool where scores above 10 denote excessive daytime sleepiness [32]. Overall illness severity was rated using the Clinical Global Impressions Severity of Illness scale (CGI-S), which provides a clinician’s assessment of the patient’s current condition [33].

Furthermore, the presence of a previously diagnosed sleep disorder and the occurrence of stressful life events prior to BMS onset were systematically recorded through patient interviews.

The full texts of all questionnaires used in the study are provided in the Supplementary Materials (Supplementary File S1).

### 2.3. Statistical Analysis

Statistical analyses were performed using IBM SPSS Statistics version 25 (IBM Corp., Armonk, NY, USA). Categorical variables were reported as frequencies and percentages and compared using Fisher’s exact test. Continuous variables were tested for normality using the Shapiro–Wilk test and described as mean ± standard deviation (SD) for normally distributed data or as median and interquartile range (IQR) for non-normally distributed data. Comparisons between groups were performed using the Mann–Whitney U test for non-normally distributed variables. Bonferroni correction was applied for multiple comparisons. A *p*-value < 0.05 was considered statistically significant, unless otherwise specified after correction.

### 2.4. Sample Size Calculation

The sample size calculation was performed using GPower 3.1.9.7 (Heinrich Heine University Düsseldorf, Düsseldorf, Germany). The expected effect size was derived from a previously published study reporting PSQI values as median and IQR in BMS patients versus controls [34]. Medians and IQRs were converted to approximate mean and standard deviation using the method described by Wan et al. (2014) [35], to enable the calculation of Cohen’s d. The effect size was then entered in G-Power to compute the required sample size for a Wilcoxon–Mann–Whitney test with two independent groups, setting the significance level (α) at 0.05 and the statistical power (1 − β) at 99%.

## 3. Results

The sample size calculation indicated that 32 participants (16 per group) were required; ultimately, 60 patients were enrolled (30 Italian [BMS-IT], 30 Romanian [BMS-RM]). The BMS-IT group included 10 males (33.3%) and 20 females (66.7%) with a mean age of 63.38 ± 10.40 years, while the BMS-RM group included 4 males (13.3%) and 26 females (86.7%) with a mean age of 58.52 ± 14.05 years.

No significant differences were found in sex distribution, age, or BMI between groups. However, BMS-IT patients had significantly fewer years of education (9.76 ± 4.29 vs. 14.07 ± 4.59 years, *p* = 0.001), while physical activity was more frequently reported by BMS-RM patients (53.3% vs. 16.7%, *p* = 0.002). Marital status, employment, smoking habits, and alcohol consumption did not differ significantly (Table 1).

The mean duration of symptoms before diagnosis was similar (BMS-IT: 15.83 ± 14.49 and BMS-RM: 16.31 ± 18.01 months, *p* = 0.911). However, BMS-RM patients consulted significantly more specialists before diagnosis (4.38 ± 1.97 vs. 2.93 ± 1.41, *p* = 0.002), were more often referred to physicians (*p* = 0.008), and were less likely to receive a correct diagnosis at the first visit (*p* = 0.005) (Table 2).

Burning sensations were the most frequently reported symptom in both groups. However, oral dysmorphism (*p* = 0.002), tingling sensation (*p* = 0.011), and allodynia (*p* = 0.023) were significantly more prevalent among BMS-RM. While the tongue was the most affected site in both cohorts, involvement of the labial commissure was more frequent in BMS-RM (*p* = 0.008). Regarding symptom patterns, BMS-RM patients more frequently reported worsening in the morning (*p* = 0.019), symptom improvement with meals (*p* = 0.001), and fewer cases with no circadian variation (*p* = 0.018) (Table 3).

**Figure 1:**
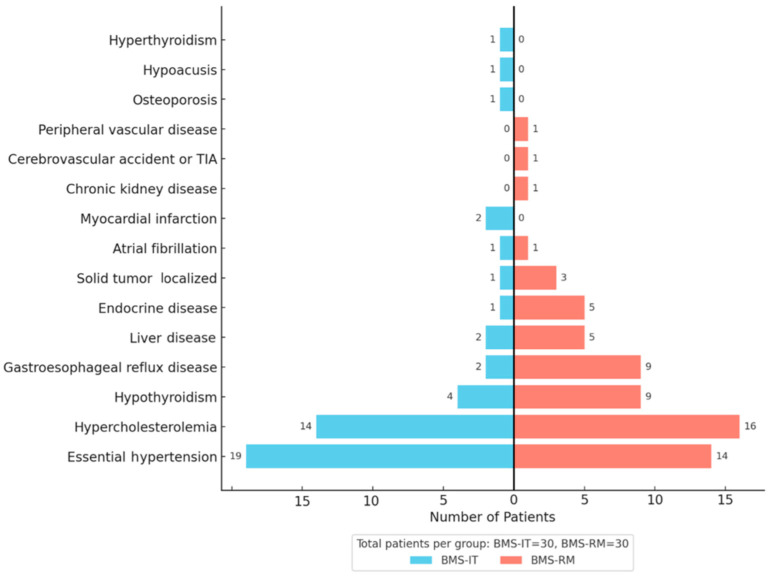
Distribution of comorbidities in BMS-IT and BMS-RM patients. The figure displays the frequency of the following comorbidities in BMS-IT (light blue) and BMS-RM (red) patients: essential hypertension, hypercholesterolemia, gastroesophageal reflux disease, localized solid tumor, liver disease, hypothyroidism, myocardial infarction, atrial fibrillation, hyperthyroidism, hypoacusis, osteoporosis, peripheral vascular disease, cerebrovascular accident or transient ischemic attack, chronic kidney disease, and endocrine disease. A butterfly chart is used for visualization.

Systemic comorbidities were broadly similar, though gastroesophageal reflux disease was more common in BMS-R patients (*p* = 0.045), and the AACCI was significantly higher in this group (median 2 vs. 0, *p* < 0.001). BMS-IT reported greater use of antiplatelet agents and angiotensin receptor blockers (*p* = 0.006 for both) (Table 4). Figure 1 schematically represents the distribution of comorbidities recorded in the BMS-IT and BMS-RM groups.

BMS-IT patients had higher pain intensity (10 [9,10] vs. 7 [5–9], *p* < 0.001)), poorer sleep quality (PSQI 9 [8–10] vs. 4 [2–6], *p* = 0.001), higher daytime sleepiness (ESS: 8 [6–10] vs. 5 [4–7], *p* = 0.001), and shorter sleep duration (5 [4–6] vs. 7 [6–8] hours/night, *p* < 0.001) with more frequent history of previous sleep disorders (66.7% vs. 16.7%, *p* < 0.001).

BMS-RM patients exhibited significantly higher anxiety scores (HAM-A: 25 [20–30] vs. 15 [13–18], *p* < 0.1) and more frequently reported stressful events preceding BMS onset (60.0% vs. 16.7%, *p* < 0.001).

**Figure 2:**
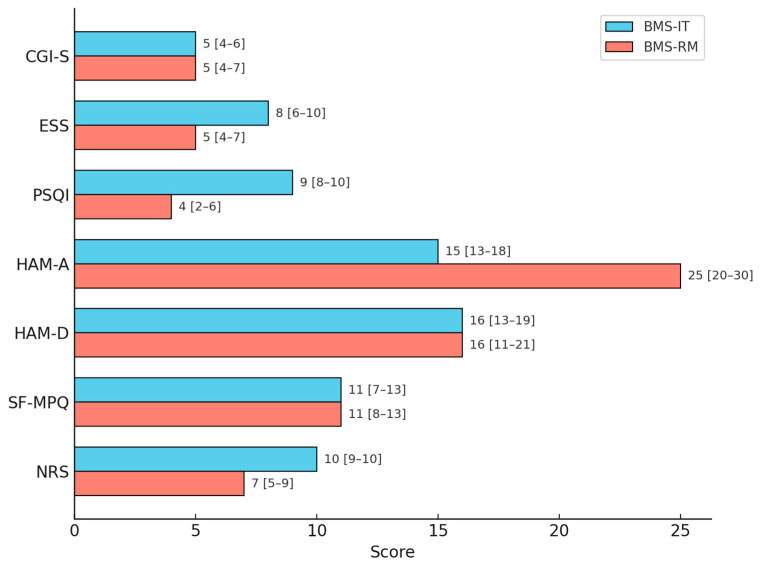
Median scores and interquartile ranges of clinical parameters in BMS-IT and BMS-RM groups. The figure displays the median values and interquartile ranges for the following variables in BMS-IT (light blue) and BMS-RM (red): Numerical Rating Scale (NRS), Short-Form McGill Pain Questionnaire (SF-MPQ), Hamilton Depression Rating Scale (HAM-D), Hamilton Anxiety Rating Scale (HAM-A), Pittsburgh Sleep Quality Index (PSQI), Epworth Sleepiness Scale (ESS), and Clinical Global Impression Severity Scale (CGI-S). A horizontal bar chart is used for visualization.

Pain quality (SF-MPQ), depression scores (HAM-D), and global severity (CGI-S) did not differ significantly. Moreover, no significant differences were found regarding psychiatric history (Table 5). Figure 2 displays the median scores and IQRs of clinical parameters for the BMS-IT and BMS-RM groups.

## 4. Discussion

This cross-national study comparing BMS patients from Italy (BMS-IT) and Romania (BMS-RM) revealed significant differences in sociodemographic characteristics, symptom profiles, psychological distress, sleep disturbances, and diagnostic pathways, highlighting the importance of cultural and healthcare contextualization in orofacial pain research. BMS is increasingly recognized as a multifactorial disorder involving complex interactions among peripheral neuropathy, central sensitization, and psychosocial influences [6]. However, few studies have systematically investigated how these dimensions may vary across populations.

BMS-IT patients had significantly fewer years of education, which may reflect broader socioeconomic disparities and potentially contribute to differences in health behaviors such as physical activity [36]. Nevertheless, despite higher educational attainment, which typically enables patients to recognize symptoms earlier and navigate the healthcare system more effectively [37], BMS-RM patients consulted a greater number of specialists and were less likely to receive a correct diagnosis at the first visit. This paradox could reflect possible structural and cultural barriers within the Romanian healthcare system, such as limited access to oral medicine specialists, the absence of standardized referral pathways, and a potentially lower integration of orofacial pain education in primary care. Moreover, cultural attitudes toward psychological symptoms and a reduced availability of multidisciplinary pain clinics may contribute to a pattern of repeated consultations before an accurate diagnosis is achieved [38]. The higher anxiety levels in BMS-RM patients may also have contributed to increased healthcare utilization, consistent with evidence linking health anxiety to more frequent consultations and amplified symptom perception [39].

Regarding symptom expression, BMS-RM patients report more frequent dysesthetic symptoms (tingling, allodynia) and perceptual disturbances such as oral dysmorphism. Notably, the only significant difference in symptom pattern was that BMS-RM patients more often experienced improvement with meals. This finding may be influenced by contextual or individual differences in symptom perception, although no direct conclusions can be drawn.

Higher pain and poorer sleep in BMS-IT may suggest a distinct psychosomatic profile that requires targeted clinical attention. These findings support the well-established bidirectional relationship between chronic pain and poor sleep [40,41]. However, it should be emphasized that these differences do not imply that BMS-IT and BMS-RM represent distinct clinical entities but rather that varying combinations of psychological burden, health behaviors, and contextual factors may influence disease severity and the lived experience of symptoms. Conversely, BMS-RM patients showed a higher prevalence of stressful life events and elevated anxiety, reinforcing the importance of psychosocial contributors to symptom persistence in this group [13].

These findings have important implications for clinical management. Tailoring treatment strategies to individual patient profiles, including psychological burden, sleep quality, and life stressors, may enhance therapeutic outcomes [42]. In this context, while traditional pharmacological and psychological approaches remain the mainstay, emerging interventions such as medical cannabis are being explored for their potential to modulate chronic pain and central sensitization [43]. Further studies are needed to clarify their efficacy and safety in BMS populations.

Although the overall burden of systemic diseases was similar, BMS-RM patients had higher AACCI scores, findings that align with recent evidence underscoring the role of non-communicable diseases and comorbidities in BMS risk and severity [22].

Importantly, despite the more structured clinical pathways and a broader diffusion of orofacial pain education in Italy, diagnostic delays remain substantial and highlight that clinician training is still insufficient to ensure early recognition and management. Supporting this, a recent large-scale analysis of diagnostic delay in 500 BMS patients found that limited specialist expertise can prolong diagnostic timelines by more than two years on average [19].

These findings indicate that the BMS clinical phenotype is shaped by an interplay of neurobiological predisposition, psychological profile, and healthcare system organization. In BMS-RM, elevated anxiety, more complex diagnostic trajectories, and frequent dysesthetic–perceptual symptoms likely reflect a stronger psychosomatic contribution and different help-seeking behaviors.

The recent literature highlights that BMS patients with elevated anxiety and somatization scores often display exaggerated cortical responses to sensory stimuli and may benefit from cognitive–behavioral or serotonergic-based interventions [44].

In BMS-IT, higher pain severity and sleep dysfunction appear more prominent but should be interpreted as reflecting a combination of factors rather than a fundamentally different pathophysiological mechanism.

This study has limitations. First, the relatively small sample size may limit the generalizability and statistical power of the findings. No significant differences were observed between the BMS-IT and BMS-RM groups in terms of sex distribution; however, both cohorts showed a marked predominance of female participants, in line with the well-established epidemiological profile of BMS reported in the literature. This female preponderance may reflect underlying hormonal, psychosocial, or neurobiological factors and underscores the need for further research into sex-related differences in disease mechanisms and therapeutic strategies [45]. Second, patients were recruited exclusively from specialized urban academic clinics. This may have introduced selection bias, potentially favoring more severe or complex cases and limiting the applicability of the findings to community-based or milder BMS populations. Furthermore, the setting in urban tertiary care centers may not accurately reflect the diagnostic and therapeutic experiences of patients in rural or underserved areas, where access to specialized care remains limited. These disparities may influence diagnostic delays and care quality. Future research should aim to include patients from diverse healthcare settings and evaluate whether digital health tools or telemedicine may reduce barriers to diagnosis and management in geographically isolated populations [46]. Third, although validated assessment tools were employed, all clinical and psychological data were self-reported, introducing potential recall or response biases. Fourth, while psychological and sleep assessments were performed using standardized instruments by trained clinicians in each country, no formal inter-rater reliability testing was conducted across centers. In addition, no blinding procedures were implemented between countries. These limitations, mainly due to the need to conduct evaluations in the participants’ native language and ensure cultural-linguistic appropriateness, may have introduced observer variability or bias. Future cross-national studies should incorporate structured inter-rater calibration and centralized rating procedures to enhance consistency and objectivity. Fifth, neuroimaging and biological marker data assessments were not conducted. Including these modalities in future research could help identify objective biological correlates and provide mechanistic insights into the observed clinical differences. Finally, while this study advances understanding of cross-cultural variation in BMS, limited engagement with the broader cross-cultural pain or psychosomatic literature may constrain the general interpretive value of the findings. Future investigations should integrate comparative frameworks from other chronic pain conditions to enrich the contextualization of cultural and healthcare system influences.

## 5. Conclusions

This cross-sectional comparison of Italian and Romanian BMS patients revealed distinct differences in pain severity, sleep quality, anxiety levels, and diagnostic trajectories. Italian patients reported more intense pain and greater sleep disturbances, while Romanian patients showed higher anxiety scores, more frequent perceptual symptoms, and longer diagnostic delays. These findings suggest that psychological burden, healthcare system factors, and sociocultural elements may influence how BMS manifests and is managed. Given these differences, clinical assessments should include routine screening for sleep quality, anxiety symptoms, and recent life stressors, which may inform personalized care plans. Moreover, the longer diagnostic pathways observed in Romanian patients highlight the need to improve early recognition in settings with limited access to oral medicine expertise. Future studies should include larger, more diverse populations and consider integrating objective biological measures (e.g., neuroimaging, biomarkers) to clarify mechanisms underlying symptom variability. Including rural and underserved populations may also help determine whether digital health or telemedicine approaches can mitigate access-related disparities in diagnosis and management.

## Figures and Tables

**Table 1:** Comparison of demographic and lifestyle variables between Italian and Romanian BMS patients.

Demographic Variables	BMS-It	BMS-RM	
**Gender**	**Frequency (%)**	**Frequency (%)**	***p*-Value**
Male	10 (33.3)	4 (13.3)	0.066
Female	20 (66.7)	26 (86.7)	
**Age (In Years)**	**Mean ± SD**	**Mean ± SD**	
	63.38 ± 10.40	58.52 ± 14.05	0.140
**BMI**	**Mean ± SD**	**Mean ± SD**	
Total	26.43 ± 3.50	25.59 ± 4.86	0.453
**Education (In Years)**	**Mean ± SD**	**Mean ± SD**	***p*-Value**
	9.76 ± 4.29	14.07 ± 4.59	0.001 *
**Family Situation**	**Frequency (%)**	**Frequency (%)**	***p*-Value ^a^**
Married	23 (73.3)	22 (70.0)	1.000
Single	3 (10.0)	1 (3.3)	0.611
Divorced	1 (3.3)	2 (6.7)	0.998
Widowed	3 (10.0)	5 (16.7)	0.706
**Employment**	**Frequency (%)**	**Frequency (%)**	***p*-Value ^b^**
Retired	16 (53.3)	16 (53.3)	1.000
Employed	6 (20.0)	9 (30.0)	0.550
Unemployed	8 (26.7)	5 (17.2)	0.530
**Physical Activity**	**Frequency (%)**	**Frequency (%)**	***p*-Value**
Yes	5 (16.7)	16 (53.3)	0.002 *
No	25 (83.3)	14 (46.7)	
**Risk Factors**			
**Smoking**	**Frequency (%)**	**Frequency (%)**	***p*-Value ^c^**
Smokers	10 (33.3)	6 (20.0)	0.379
Never Smokers	20 (66.7)	24 (80.0)	
Very Light Smokers (<5 Cigarettes)	2 (6.7)	3 (10.0)	0.640
Light Smokers (5–10 Cigarettes)	3 (10.3)	0	0.075
Moderate Smokers (10–15 Cigarettes)	2 (6.7)	2 (6.7)	1.000
Heavy Smokers (>15 Cigarettes)	3 (10.3)	0	0.075
E-Cig	0	1 (3.3)	0.313
Heat-Not-Burn	0	0	-
**Alcohol Use**	**Frequency (%)**	**Frequency (%)**	***p*-Value ^d^**
Drinkers	6 (20.0)	4 (13.3)	0.730
Not Drinkers	24 (80.0)	26 (86.7)	
Light/Moderate Drinkers (≤14 Units/Week)	5 (16.7)	4 (13.3)	1.000
Heavy Drinkers (>14 Units/Week)	1 (3.3)	0	1.000

A significant difference between the percentages is measured by Fisher’s exact test. The significant difference between means is measured by the Mann–Whitney U test. * Statistically significant values. ^a^ Family situation is considered significant with a Bonferroni correction of 0.0167. ^b^ Employment status is considered significant with a Bonferroni correction of 0.0125. ^c^ Smoking habits are considered significant with a Bonferroni correction of 0.01. ^d^ Alcohol use is considered significant with a Bonferroni correction of 0.025. All *p*-values reported as significant are compared against Bonferroni-adjusted significance thresholds. Abbreviation: BMI, body mass index; BMS-IT, Burning Mouth Syndrome—Italian; and BMS-RM, Burning Mouth Syndrome—Romanian.

**Table 2:** Diagnostic history and referral patterns in BMS Patients: Italy vs. Romania.

	BMS-IT	BMS-RM	
**Diagnostic History**	**Mean ± SD**	**Mean ± SD**	***p*** **-Value**
Disease onset (months)	15.83 ± 14.49	16.31 ± 18.01	0.911
Number of doctors consulted prior to diagnosis of BMS	2.93 ± 1.412	4.38 ± 1.971	0.002 *
**Referrals**	**Frequency (%)**	**Frequency (%)**	***p*** **-Value ^a^**
Dentist	23 90 (76.7)	22 (73.3)	0.551
Physician	18 (60.0)	10 (33.3)	0.008
Maxillofacial surgeon	6 (20.0)	9 (30.0)	0.753
Otolaryngologist	10 (33.33)	16 (53.3)	0.193
Gastroenterologist	11(36.67)	19 (63.3)	0.071
Neurologist	3 (10.0)	8 (26.67)	0.182
Psychiatrist	3 (10.0)	6 (20.0)	0.470
**Diagnosis Experience**	**Frequency (%)**	**Frequency (%)**	***p*** **-Value ^b^**
No diagnosis	0 (0.00)	7 (0.23)	0.005 *
Diagnosis right the first time	3 (10.0)	3 (10.0)	1.000
	**Median [IQR]**	**Median [IQR]**	***p*** **-Value**
Number of misdiagnoses	3.0 [2, 3]	3.0 [2–4]	0.203

A significant difference between the percentages is measured by Fisher’s exact test. The significant difference between means is measured by the Mann–Whitney U test. The significant difference between medians is measured by the Mann–Whitney U test. * Statistically significant values. ^a^ The type of doctors consulted is considered significant with a Bonferroni correction of 0.007. ^b^ Diagnosis experience is considered significant with a Bonferroni correction of 0.025. All *p*-values reported as significant are compared against Bonferroni-adjusted significance thresholds. Abbreviation: BMS-IT, Burning Mouth Syndrome—Italian; and BMS-RM, Burning Mouth Syndrome—Romanian.

**Table 3:** Clinical symptoms, localization, and temporal patterns in BMS patients: Italy vs. Romania.

	BMS-IT	BMS-RM	
**Symptoms**	**Frequency (%)**	**Frequency (%)**	***p*-Value ^a^**
Burning	30 (100)	30 (100)	1.000
Xerostomia	17 (56.7)	15 (50.0)	0.792
Dysgeusia	11 (36.7)	8 (26.7)	0.576
Globus pharyngeus	10 (33.3)	13 (43.3)	0.591
Oral dysmorphism	5 (16.7)	14 (46.7)	0.002 *
Sialorrhea	9 (30.0)	9 (30.0)	1.000
Tingling sensation	4 (13.3)	14 (46.7)	0.011
Oral foreign body sensation	8 (26.7)	11 (36.7)	0.576
Occlusal dysesthesia	4 (13.3)	6 (20.0)	0.728
Oral dyskinesia	1 (3.3)	0	0.999
Hypoesthesia	3 (10.0)	0	0.236
Subjective halitosis	0	3 (10.0)	0.075
Dysosmia	1 (3.3)	1 (3.3)	1.000
Itching	7 (23.3)	9 (30.0)	0.769
Allodynia	2 (6.7)	10 (33.3)	0.023
**Worst symptoms**	**Frequency (%)**	**Frequency (%)**	***p*-Value ^b^**
Burning	27 (90)	8 (26.7)	0.0001 *
Oral dysmorphism	1 (3.3)	0	1.000
Xerostomia	2 (6.7)	13 (43.3)	0.370
Tingling sensation	0	5 (16.7)	1.000
Dysgeusia	0	1 (3.3)	0.009
Globus pharyngeus	0	2 (6.7)	1.000
Itching	0	1 (3.3)	1.000
**Localization**	**Frequency (%)**	**Frequency (%)**	***p*-Value ^c^**
Tongue	26 (86.7)	23 (76.7)	0.277
Lips	21 (70.0)	16 (53.3)	0.172
Palate	17 (56.7)	16 (53.3)	0.791
Gums	18 (60.0)	12 (40.0)	0.115
Labial commissure	17 (56.7)	7 (23.3)	0.008
Cheeks	18 (60.0)	11 (36.7)	0.066
Floor of the mouth	18 (60.0)	16 (53.3)	0.594
Retromolar trigone	14 (46.7)	11 (36.7)	0.426
**Symptom Pattern**	**Frequency (%)**	**Frequency (%)**	***p*-Value ^d^**
Worse in the evening	11 (36.7)	11 (36.7)	1.000
Worse in the morning	0	5 (16.7)	0.019
No circadian changes	19 (63.3)	10 (33.3)	0.018
Continuous	12 (40.0)	14 (46.7)	0.162
Intermittent	18 (60.0)	16 (53.3)	0.430
Improve with meals	2 (6.7)	14 (46.7)	0.001 *

A significant difference between the percentages is measured by Fisher’s exact test. * Statistically significant values. ^a^ Symptoms are considered significant with a Bonferroni correction of 0.0033. ^b^ Localization is considered significant with a Bonferroni correction of 0.006. ^c^ The worst symptoms are considered significant with a Bonferroni correction of 0.007. ^d^ Symptom pattern is considered significant with a Bonferroni correction of 0.008. All *p*-values reported as significant are compared against Bonferroni-adjusted significance thresholds. Abbreviation: BMS-IT, Burning Mouth Syndrome–Italian; and BMS-RM, Burning Mouth Syndrome–Romanian.

**Table 4:** The prevalence of systemic diseases, Age-Adjusted Charlson Comorbidity Index, and the drug consumption of samples of patients.

	BMS-IT	BMS-RM	
**Systemic Diseases**	**Frequency (%)**	**Frequency (%)**	***p*-Value ^a^**
Essential hypertension	19 (63.3)	14 (46.7)	0.299
Hypercholesterolemia	14 (46.7)	16 (53.3)	0.796
Gastroesophageal reflux disease	2 (6.7)	9 (30.0)	0.045
Solid tumor localized	1 (3.3)	3 (10.0)	0.605
Liver disease	2 (6.7)	5 (16.7)	0.421
Hypothyroidism	4 (13.3)	9 (30.0)	0.210
Myocardial infarction	2 (6.7)	0	0.472
Atrial fibrillation	1 (3.3)	1 (3.3)	1.000
Hyperthyroidism	1 (3.3)	0	1.000
Hypoacusis	1 (3.3)	0	1.000
Osteoporosis	1 (3.3)	0	1.000
Peripheral vascular disease	0	1 (3.3)	1.000
Cerebrovascular accident or TIA	0	1 (3.3)	1.000
Chronic kidney disease	0	1 (3.3)	1.000
Endocrine disease	1 (3.3)	5 (16.7)	0.197
**Age-Adjusted Charlson Comorbidity Index**	**Median [IQR]**	**Median [IQR]**	***p*-Value**
AACCI	0 [0, 1]	2 [1–3]	<0.001 *
**Drug Consumption**	**Frequency (%)**	**Frequency (%)**	***p*-Value ^b^**
Antiplatelets	12 (40.0)	2 (6.7)	0.006
Angiotensin receptor blockers	12 (40.0)	2 (6.7)	0.006
Statins	7 (23.3)	10 (33.3)	0.567
Beta blockers	6 (20.0)	2 (6.7)	0.255
ACE-inhibitors	6 (20.0)	5 (16.7)	1.000
Levothyroxine	5 (16.7)	2 (6.7)	0.421
Diuretics	4 (13.3)	5 (16.7)	1.000
Proton pump inhibitors	3 (10.0)	6 (20.0)	0.469
Calcium channel blockers	1 (3.3)	2 (6.7)	1.000
Blood thinners	1 (3.3)	0	1.000
Ezetimibe	0 (0.0)	2 (6.7)	0.472

A significant difference between the percentages is measured by Fisher’s exact test. The significant difference between medians is measured by the Mann–Whitney U test. * Statistically significant values. ^a^ Comorbidities are considered significant with a Bonferroni correction of 0.003. ^b^ Drugs are considered significant with a Bonferroni correction of 0.005. All *p*-values reported as significant are already adjusted using Bonferroni correction. Abbreviation: ACCI, Age-Adjusted Charlson Comorbidity Index; BMS-IT, Burning Mouth Syndrome–Italian; and BMS-RM, Burning Mouth Syndrome–Romanian.

**Table 5:** Clinical, sleep, and psychological differences in sample patients.

	BMS-IT	BMS-RM	
**Clinical Parameter**	**Median [IQR]**	**Median [IQR]**	***p*** **-Value ^a^**
**Pain**			
NRS	10 [9, 10]	7 [5–9]	<0.001 *
SF-MPQ **Psychological assessment**	11 [7–13]	11 [8–13]	0.887
HAM-D	16 [13–19]	16 [11–21]	0.984
HAM-A	15 [13–18]	25 [20–30]	<0.001 *
CGI-S	5 [4–6]	5 [4–7]	0.230
**Sleep**			
PSQI	9 [8–10]	4 [2–6]	0.001 *
ESS	8 [6–10]	5 [4–7]	0.001 *
Sleep duration (hours/night)	5 [4–6]	7 [6–8]	<0.001 *
**History**	**Frequency (%)**	**Frequency (%)**	***p*** **-Value ^b^**
Previous sleep disorder	20 (66.7)	5 (16.7)	<0.001 *
Psychiatric history	9 (30.0)	4 (13.3)	0.115
Reports stressful life event preceding BMS onset	5 (16.7)	18 (60.0)	<0.001 *

A significant difference between the percentages is measured by Fisher’s exact test. IQR is the interquartile range. The significant difference between medians is measured by the Mann–Whitney U test. * Statistically significant values. ^a^ A significant level of clinical parameters is considered with a Bonferroni correction of 0.007. ^b^ A significant level of history parameters is considered with a Bonferroni correction of 0.017. All *p*-values reported as significant are compared against Bonferroni-adjusted significance thresholds. Abbreviation: NRS, Numeric Rating Scale for pain; SF-MPQ, Short form of McGill Pain Questionnaire; HAM-D; Hamilton Depression Rating Scale; HAM-A, Hamilton Anxiety Rating Scale; CGI-S, Clinical Global Impression—Severity; PSQI, Pittsburgh Sleep Quality Index; ESS, Epworth Sleepiness Scale; BMS-IT, Burning Mouth Syndrome—Italian; and BMS-RM, Burning Mouth Syndrome—Romanian.

## Data Availability

The original contributions presented in the study are included in the article, further inquiries can be directed to the corresponding authors (Gennaro Musella, gennaro.musella@unifg.it).

## Supplementary Materials

The following supporting information can be downloaded at: https://www.mdpi.com/article/10.3390/jcm14165805/s1.

## Abbreviations

The following abbreviations are used in this manuscript:

AACCI	Age-Adjusted Charlson Comorbidity Index
BMS	Burning Mouth Syndrome
BMI	Body Mass Index
CGI-S	Clinical Global Impressions Severity of Illness Scale
ESS	Epworth Sleepiness Scale
GERD	Gastroesophageal Reflux Disease
HAM-A	Hamilton Anxiety Rating Scale
HAM-D	Hamilton Depression Rating Scale
ICOP	International Classification of Orofacial Pain
IASP	International Association for the Study of Pain
IQR	Interquartile Range
NRS	Numeric Rating Scale
OSAS	Obstructive Sleep Apnea Syndrome
PSQI	Pittsburgh Sleep Quality Index
SF-MPQ	Short Form McGill Pain Questionnaire
STROBE	Strengthening the Reporting of Observational Studies in Epidemiology
