# Supplementary material for: Cross-Cultural Differences and Clinical Presentations in Burning Mouth Syndrome: A Cross-Sectional Comparative Study of Italian and Romanian Outpatient Settings
Source: J Clin Med. 2025 Aug 16;14(16):5805. doi: 10.3390/jcm14165805 (PMC12387540; doi:10.3390/jcm14165805)
Supplement: Supplementary file 1 [file jcm-14-05805-s001.zip › jcm-3772118-supplementary.pdf]

SUPPLEMENTARY FILE S1 – FULL TEXT OF QUESTIONNAIRES USED IN THE STUDY

This file includes the full versions of the self-administered and clinician-rated questionnaires employed in the present study:

1. **Numeric Rating Scale (NRS)**
2. **Hamilton Anxiety Rating Scale (HAM-A)**
3. **Hamilton Depression Rating Scale (HAM-D)**
4. **Pittsburgh Sleep Quality Index (PSQI)**
5. **Epworth Sleepiness Scale (ESS)**
6. **Short-Form McGill Pain Questionnaire**
7. **Clinical Global Impression – Severity (CGI-S)**

1. Numeric Rating Scale (NRS)

Reference: Hawker, G.A.; Mian, S.; Kendzerska, T.; French, M. Measures of adult pain: Visual Analog Scale for Pain (VAS Pain), Numeric Rating Scale for Pain (NRS Pain), McGill Pain Questionnaire (MPQ), Short-Form McGill Pain Questionnaire (SF-MPQ), Chronic Pain Grade Scale (CPGS), Short Form-36 Bodily Pain Scale (SF-36 BPS), and Measure of Intermittent and Constant Osteoarthritis Pain (ICOAP). Arthritis Care Res (Hoboken) 2011, 63 Suppl 11, S240-252, doi:10.1002/acr.20543.

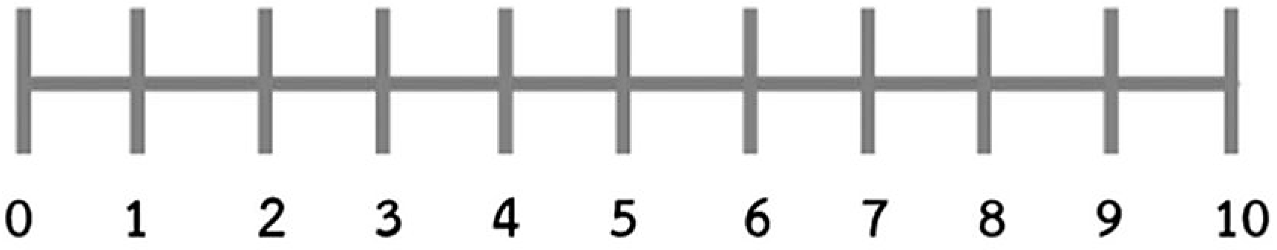

2. Hamilton Anxiety Rating Scale (HAM-A)

Reference: Hamilton M.The assessment of anxiety states by rating. Br J Med Psychol 1959; 32:50–55.

Hamilton Anxiety Rating Scale (HAM-A)

Below is a list of phrases that describe certain feeling that people have. Rate the patients by finding the answer which best describes the extent to which he/she has these conditions. Select one of the five responses for each of the fourteen questions.

0 = Not present, 1 = Mild, 2 = Moderate, 3 = Severe, 4 = Very severe.

1 Anxious mood [0] [1] [2] [3] [4]

Worries, anticipation of the worst, fearful anticipation, irritability.

2 Tension [0] [1] [2] [3] [4]

Feelings of tension, fatigability, startle response, moved to tears easily, trembling, feelings of restlessness, inability to relax.

3 Fears [0] [1] [2] [3] [4]

Of dark, of strangers, of being left alone, of animals, of traffic, of crowds.

4 Insomnia [0] [1] [2] [3] [4]

Difficulty in falling asleep, broken sleep, unsatisfying sleep and fatigue on waking, dreams, nightmares, night terrors.

5 Intellectual [0] [1] [2] [3] [4]

Difficulty in concentration, poor memory.

6 Depressed mood [0] [1] [2] [3] [4]

Loss of interest, lack of pleasure in hobbies, depression, early waking, diurnal swing.

7 Somatic (muscular) [0] [1] [2] [3] [4]

Pains and aches, twitching, stiffness, myoclonic jerks, grinding of teeth, unsteady voice, increased muscular tone.

8 Somatic (sensory) [0] [1] [2] [3] [4]

Tinnitus, blurring of vision, hot and cold flushes, feelings of weakness, pricking sensation.

9 Cardiovascular symptoms [0] [1] [2] [3] [4]

Tachycardia, palpitations, pain in chest, throbbing of vessels, fainting feelings, missing beat.

10 Respiratory symptoms [0] [1] [2] [3] [4]

Pressure or constriction in chest, choking feelings, sighing, dyspnea.

11 Gastrointestinal symptoms [0] [1] [2] [3] [4]

Difficulty in swallowing, wind abdominal pain, burning sensations, abdominal fullness, nausea, vomiting, borborygmi, looseness of bowels, loss of weight, constipation.

12 Genitourinary symptoms [0] [1] [2] [3] [4]

Frequency of micturition, urgency of micturition, amenorrhea, menorrhagia, development of frigidity, premature ejaculation, loss of libido, impotence.

13 Autonomic symptoms [0] [1] [2] [3] [4]

Dry mouth, flushing, pallor, tendency to sweat, giddiness, tension headache, raising of hair.

14 Behavior at interview [0] [1] [2] [3] [4]

Fidgeting, restlessness or pacing, tremor of hands, furrowed brow, strained face, sighing or rapid respiration, facial pallor, swallowing, etc.

3. Hamilton Depression Rating Scale (HAM-D)

Reference: Hamilton M. A rating scale for depression. J Neurol Neurosurg Psychiatry 1960; 23:56–62

Hamilton Depression Rating Scale (HDRS)

PLEASE COMPLETE THE SCALE BASED ON A STRUCTURED INTERVIEW

Instructions: for each item select the one "cue" which best characterizes the patient. Be sure to record the answers in the appropriate spaces (positions 0 through 4).

1 DEPRESSED MOOD *(sadness, hopeless, helpless, worthless)*
- 0 |__| Absent.
- 1 |__| These feeling states indicated only on questioning.
- 2 |__| These feeling states spontaneously reported verbally.
- 3 |__| Communicates feeling states non-verbally, i.e. through facial expression, posture, voice and tendency to weep.
- 4 |__| Patient reports virtually only these feeling states in his/her spontaneous verbal and non-verbal communication.

3 SUICIDE
- 0 |__| Absent.
- 1 |__| Feels life is not worth living.
- 2 |__| Wishes he/she were dead or any thoughts of possible death to self.
- 3 |__| Ideas or gestures of suicide.
- 4 |__| Attempts at suicide (any serious attempt rate 4).

4 INSOMNIA: EARLY IN THE NIGHT
- 0 |__| No difficulty falling asleep.
- 1 |__| Complains of occasional difficulty falling asleep, i.e. more than ½ hour.
- 2 |__| Complains of nightly difficulty falling asleep.

5 INSOMNIA: MIDDLE OF THE NIGHT
- 0 |__| No difficulty.
- 1 |__| Patient complains of being restless and disturbed during the night.
- 2 |__| Waking during the night – any getting out of bed rates 2 (except for purposes of voiding).

6 INSOMNIA: EARLY HOURS OF THE MORNING
- 0 |__| No difficulty.
- 1 |__| Waking in early hours of the morning but goes back to sleep.
- 2 |__| Unable to fall asleep again if he/she gets out of bed.

7 WORK AND ACTIVITIES
- 0 |__| No difficulty.
- 1 |__| Thoughts and feelings of incapacity, fatigue or weakness related to activities, work or hobbies.
- 2 |__| Loss of interest in activity, hobbies or work – either directly reported by the patient or indirect in listlessness, indecision and vacillation (feels he/she has to push self to work or activities).
- 3 |__| Decrease in actual time spent in activities or decrease in productivity. Rate 3 if the patient does not spend at least three hours a day in activities (job or hobbies) excluding routine chores.
- 4 |__| Stopped working because of present illness. Rate 4 if patient engages in no activities except routine chores, or if patient fails to perform routine chores unassisted.

8 RETARDATION (slowness of thought and speech, impaired ability to concentrate, decreased motor activity)
- 0 |__| Normal speech and thought.
- 1 |__| Slight retardation during the interview.
- 2 |__| Obvious retardation during the interview.
- 3 |__| Interview difficult.
- 4 |__| Complete stupor.

9 AGITATION
- 0 |__| None.
- 1 |__| Fidgetiness.
- 2 |__| Playing with hands, hair, etc.
- 3 |__| Moving about, can't sit still.
- 4 |__| Hand wringing, nail biting, hair-pulling, biting of lips.

10 ANXIETY PSYCHIC
- 0 |__| No difficulty.
- 1 |__| Subjective tension and irritability.
- 2 |__| Worrying about minor matters.
- 3 |__| Apprehensive attitude apparent in face or speech.
- 4 |__| Fears expressed without questioning.

2 FEELINGS OF GUILT
- 0 |__| Absent.
- 1 |__| Self reproach, feels he/she has let people down.
- 2 |__| Ideas of guilt or rumination over past errors or sinful deeds.
- 3 |__| Present illness is a punishment. Delusions of guilt.
- 4 |__| Hears accusatory or denunciatory voices and/or experiences threatening visual hallucinations.

11 ANXIETY SOMATIC (physiological concomitants of anxiety) such as:
gastro-intestinal – dry mouth, wind, indigestion, diarrhea, cramps, belching
cardio-vascular – palpitations, headaches
respiratory – hyperventilation, sighing
urinary frequency
sweating
- 0 |__| Absent.
- 1 |__| Mild.
- 2 |__| Moderate.
- 3 |__| Severe.
- 4 |__| Incapacitating.

12 SOMATIC SYMPTOMS GASTRO-INTESTINAL
- 0 |__| None.
- 1 |__| Loss of appetite but eating without staff encouragement. Heavy feelings in abdomen.
- 2 |__| Difficulty eating without staff urging. Requests or requires laxatives or medication for bowels or medication for gastro-intestinal symptoms.

13 GENERAL SOMATIC SYMPTOMS
- 0 |__| None.
- 1 |__| Heaviness in limbs, back or head. Backaches, headaches, muscle aches. Loss of energy and fatigability.
- 2 |__| Any clear-cut symptom rates 2.

14 GENITAL SYMPTOMS (symptoms such as loss of libido, menstrual disturbances)
- 0 |__| Absent.
- 1 |__| Mild.
- 2 |__| Severe.

15 HYPOCHONDRIASIS
- 0 |__| Not present.
- 1 |__| Self-absorption (bodily).
- 2 |__| Preoccupation with health.
- 3 |__| Frequent complaints, requests for help, etc.
- 4 |__| Hypochondriacal delusions.

16 LOSS OF WEIGHT *(RATE EITHER a OR b)*

a) According to the patient:	b) According to weekly measurements:
0 |__| No weight loss.	0 |__| Less than 1 lb weight loss in week.
1 |__| Probable weight loss associated with present illness.	1 |__| Greater than 1 lb weight loss in week.
2 |__| Definite (according to patient) weight loss.	2 |__| Greater than 2 lb weight loss in week.
3 |__| Not assessed.	3 |__| Not assessed.

17 INSIGHT
- 0 |__| Acknowledges being depressed and ill.
- 1 |__| Acknowledges illness but attributes cause to bad food, climate, overwork, virus, need for rest, etc.
- 2 |__| Denies being ill at all.

Total score: |__|__|

4. Pittsburgh Sleep Quality Index (PSQI)

Reference: Buysse, D. J., Reynolds, C. F., 3rd, Monk, T. H., Berman, S. R., & Kupfer, D. J. (1989). The Pittsburgh Sleep Quality Index: a new instrument for psychiatric practice and research. *Psychiatry research*, *28*(2), 193–213. https://doi.org/10.1016/0165-1781(89)90047-4

PITTSBURGH SLEEP QUALITY INDEX (PSQI)

1. During the past month, when have you usually gone to bed at night?

2. During the past month, how long (in minutes) has it usually taken you to fall asleep eacl night?

3. During the past month, when have you usually gotten up in the morning?

4. During the past month, how many hours of actual sleep did you get at night? (This may be different than the number of hours you spend in bed.)

5. During the past month, how often have you had trouble sleeping because you……..

(a) cannot get to sleep within 30 minutes.
(b) wake up in the middle of the night or early morning.
(c) have to get up to use the bathroom.
(d) cannot breathe comfortably.
(e) cough or snore loudly.
(f) feel too cold.
(g) feel too hot.
(h) had bad dreams.
(i) have pain.

Not during the past month________ Less than once a week_______ Once or twice a week_______ Three or more times a week______

6. During the past month, how would you rate your sleep quality overall?

Very good _____________
Fairly good _____________
Fairly bad _____________
Very bad _____________

7. During the past month, how often have you taken medicine (prescribed or "over the counter") to help you sleep?

Not during the past month________ Less than once a week_______ Once or twice a week_______ Three or more times a week______

8. During the past month, how often have you had trouble staying awake while driving, eating meals, or engaging in social activity?

Not during the past month________ Less than once a week_______ Once or twice a week_______ Three or more times a week______

9. During the past month, how much of a problem has it been for you to keep up enough enthusiasm to get things done?

No problem at all _________
Only a very slight problem _________
Somewhat of a problem _________
A very big problem _________

5. Epworth Sleepiness Scale (ESS)

Reference: Johns M. W. (1991). A new method for measuring daytime sleepiness: the Epworth sleepiness scale. *Sleep*, *14*(6), 540–545. https://doi.org/10.1093/sleep/14.6.540

EPWORTH SLEEPINESS SCALE

	Would never nod off 0	Slight chance of nodding off 1	Moderate chance of nodding off 2	High chance of nodding off 3
**Sitting and reading**				
**Watching TV**				
**Sitting, inactive**, in a public place (e.g., in a meeting, theater, or dinner event)				
**As a passenger in a car** for an hour or more without stopping for a break				
**Lying down to rest** when circumstances permit				
**Sitting and talking** to someone				
**Sitting quietly** after a meal without alcohol				
**In a car, while stopped** for a few minutes in traffic or at a light				

6. Short-Form McGill Pain Questionnaire

Reference: Melzack R. (1987). The short-form McGill Pain Questionnaire. *Pain*, *30*(2), 191–197. https://doi.org/10.1016/0304-3959(87)91074-8

SHORT-FORM MCGILL PAIN QUESTIONNAIRE

	NONE	MILD	MODERATE	SEVERE
1. THROBBING	0) ____	1) ____	2) ____	3) ____
2. SHOOTING	0) ____	1) ____	2) ____	3) ____
3. STABBING	0) ____	1) ____	2) ____	3) ____
4. SHARP	0) ____	1) ____	2) ____	3) ____
5. CRAMPING	0) ____	1) ____	2) ____	3) ____
6. GNAWING	0) ____	1) ____	2) ____	3) ____
7. HOT-BURNING	0) ____	1) ____	2) ____	3) ____
8. ACHING	0) ____	1) ____	2) ____	3) ____
9. HEAVY	0) ____	1) ____	2) ____	3) ____
10. TENDER	0) ____	1) ____	2) ____	3) ____
11. SPLITTING	0) ____	1) ____	2) ____	3) ____
12. TIRING-EXHAUSTING	0) ____	1) ____	2) ____	3) ____
13. SICKENING	0) ____	1) ____	2) ____	3) ____
14. FEARFUL	0) ____	1) ____	2) ____	3) ____
15. PUNISHING-CRUEL	0) ____	1) ____	2) ____	3) ____

7. Clinical Global Impression – Severity (CGI-S)

Reference: Busner, J.; Targum, S.D. The clinical global impressions scale: applying a research tool in clinical practice. *Psychiatry (Edgmont)* **2007**, *4*, 28-37.

Clinical Global Impression (CGI)

1. Severity of illness
Considering your total clinical experience with this particular population, how mentally ill is the patient at this time?

0 = Not assessed	4 = Moderately ill
1 = Normal, not at all ill	5 = Markedly ill
2 = Borderline mentally ill	6 = Severely ill
3 = Mildly ill	7 = Among the most extremely ill patients
